# 

**Published:** 1892-03

**Authors:** 


VOL. XIII. MARCH, 1892. No. 3.

### THE
### International Dental Journal

A MONTHLY PERIODICAL

DEVOTED TO

### DENTAL AND ORAL SCIENCE.

JAMES TRUMAN, D.D.S., EDITOR.

JOSEPH HEAD, M.D., D.D.S., ASSISTANT EDITOR.

Publication Office,

716 FILBERT STREET, PHILADELPHIA.

PUBLISHED BY THE

INTERNATIONAL DENTAL PUBLICATION COMPANY,

**51 WEST THIRTY-SEVENTH STREET, NEW YORK CITY,**

—AND—

**716 FILBERT STREET, PHILADELPHIA.**

**Entered at the Post-Office at Philadelphia, and admitted for transmission through the mails at second-class rates.**

$2.50 per Annum, in Advance. Single Copies, 25 Cents.

PRINTED BY J. B. LIPPINCOTT COMPANY, PHILADELPHIA.

